# Clinical Use of Propranolol Reduces Biomarkers of Proliferation in Gastric Cancer

**DOI:** 10.3389/fonc.2021.628613

**Published:** 2021-04-26

**Authors:** Qian Hu, Ping Liao, Wei Li, Jiali Hu, Cuiyu Chen, Yu Zhang, Yang Wang, Ling Chen, Kun Song, Jie Liu, Wei Zhang, Qing Li, Howard L. McLeod, Yijing He

**Affiliations:** ^1^ Department of Clinical Pharmacology, Xiangya Hospital, Central South University, Changsha, China; ^2^ Institute of Clinical Pharmacology, Central South University, Hunan Key Laboratory of Pharmacogenetics, Changsha, China; ^3^ National Clinical Research Center for Geriatric Disorders, Xiangya Hospital, Central South University, Changsha, China; ^4^ Department of Pharmacy, Affiliated Hospital of Nantong University, Nantong, China; ^5^ Department of Gastrointestinal Surgery, Xiangya Hospital, Central South University, Changsha, China; ^6^ USF Taneja College of Pharmacy, Tampa, FL, United States

**Keywords:** gastric, propranolol, anti-proliferation, CD8^+^ T cell, immunity, AKT/MAPK

## Abstract

Gastric cancer has one of the highest mortality rate in the world, but the treatment is still limited. Building on previous studies, mechanistic studies on propranolol in gastric cancer mice models and gastric cancer patients were performed. Propranolol inhibited the *in vitro* proliferation of gastric cancer cells in a time- and concentration-dependent manner. Consistent findings were observed in MFC tumors engrafted 615 mice, which were treated with propranolol at 10 mg/kg daily for 14 days. Propranolol inhibited the phosphorylation of AKT, MEK, and ERK proteins than control in mice tumor tissues respectively (p-AKT 26.16 vs. 56.82, *P* = 0.0196, p-MEK 28.27 vs. 59.28, *P* = 0.1102, p-ERK 48.2 vs. 107.4, P = 0.0062). Propranolol had antiproliferative activity in gastric cancer patients receiving 60 mg daily for 7 days prior to surgery(ki67 44.8 vs 125.3 for placebo; P = 0.02). Phosphorylated AKT, MEK, and ERK did not differ between propranolol and placebo treatment in gastric cancer patients. The expression of molecules on CD8^+^ T cells was not changed both in mice model and patients nor was there a statistically significant difference in CD8^+^ T cell subsets in patients, although suggestion of an effect was evident. These results prove that propranolol may inhibit the growth of gastric cancer in mice model and patients and the possible mechanism was *via* inhibiting the AKT and MAPK pathways, but the frequency of tumor infiltration CD8^+^ T cells did not increase significantly.

## Introduction

Propranolol is a non-selective β-adrenergic receptor (β-AR) blocker, which is mainly used for hypertension and is the first-line therapy for infantile hemangioma (1). In the past decade, many studies have proved that propranolol can induce apoptosis, inhibit proliferation, angiogenesis, and metastasis across solid tumors (2–6). Recent data suggested that propranolol suppressed colorectal cancer cell growth through simultaneously activating autologous CD8^+^ T cells and decreasing the phosphorylation level of mitogen-activated protein kinase (MAPK)/(ATP-dependent tyrosine kinases) AKT pathway (7). Several studies also observed that propranolol may exert an anti-tumor effect in melanoma and breast cancer by suppressing AKT and MAPK signaling pathways (8).

Propranolol-associated suppression of tumor growth has also been associated with enhanced intratumoral antitumor immune response in breast cancer (6) and colorectal cancer (7). However, if propranolol can suppress gastric tumor growth by elevating tumor infiltration of CD8^+^ T cells is unclear. In this study, we hypothesized that propranolol may suppress the growth of gastric cancer by inhibiting the proliferation signaling *in vitro* and *in vivo*. The changes of T lymphocytes in tumor microenvironment were measured simultaneously.

## Materials and Methods

### Cell Lines and Reagents

The AGS and MFC cell lines were purchased from the Cell Bank of the Chinese Academy of Sciences (Kunming, China). The HGC-27 cell line was purchased from Cell Bank of Typical Culture Preservation Committee, Chinese Academy of Sciences (Shanghai). AGS and HGC-27 were cultured in DMEM medium (Gibco, Life Technologies, China), MFC was cultured in 1640 medium (Gibco, Life Technologies, China) supplemented with 10% FBS (Gibco, Life Technologies Australia), 100 U/ml penicillin, and 100 μg/ml streptomycin at 37°C and 5% CO2 in tissue culture incubator.

### Cell Viability Assays

The half-maximal inhibitory concentration (IC50) value was determined by CellTiter 96^®^ AQueous One Solution Cell Proliferation Assay (MTS, Promega). Cells were plated in 96-well plates at a density of 2 to 4 × 10^3^ and treated with 20 to 140 μM propranolol (propranolol hydrochloride, P0884, Sigma-Aldrich, USA) for 24, 48, and 72 h. Then, MTS assays were do according to the manufacturer’s protocols. Each test was carried out in triplicate.

### Animals and Treatments

Six weeks old 615 male mice (Institute of Transfusion and Hematology, Chinese Academy of Medical Sciences) were injected subcutaneously into the right flank with 10^6^ living MFC cells in 100 µl PBS. Tumor volume was measured two times/week and tumor volume calculated = W^2^ × L/2 mm^3^. After tumor volume reached 50 mm^3^, the mice were randomly divided into control and propranolol groups. The propranolol group received 10 mg/kg propranolol in 100 µl of PBS and control group received 100 µl PBS by intraperitoneal injection every day. Treatment continued for the duration of the experiment. The mice were sacrificed at the end of treatment (around 2 weeks) and tumors were removed for further analysis.

### Immunohistochemistry Assay

The paraffin-free tissue sections were treated with citrate antigen repair buffer (pH 6.0), and then the tissue sections were incubated with peroxidase blocking solution (S2023, Dako) for 15 min and blocked with protein (X0909, Dako) for 30 min. The corresponding specific primary antibodies were used: phosphor-ser221-MEK (rabbit, ab96379, Abcam), phospho-ERK1/2-Thr202/Tyr204 (rabbit, 4370, CST) and phospho-ser473-AKT (rabbit, 4060, CST). After treatment, the slides were incubated overnight at 4°C. Rabbit HRP conjugated secondary antibody (K4003, Dako) and hematoxylin (MHS32, Sigma) counterstain were used for treatment. The samples were observed with a digital microscope (Panoramic Viewer, 3D HISTECH), and protein expression was measured with a histochemical score (H-Score). Immunohistochemical results were scored with H-SCORE. The number of positive cells in each section H-SCORE = ∑ (PI × I) = (percentage of weak-strength cells × 1) + (percentage of medium-strength cells × 2) + percentage of cells. In this formula, PI represents the percentage of positive cells in the section, and I represents the staining intensity (9–11).

### Flow Cytometry

The mouse tumor was cut into 2- to 3-mm pieces to make a single cell suspension. The tumor and spleen were mechanically macerated and passed directly through a 70-mm nylon cell filter (Corning). The cells were washed with flowing buffer (0.1% BSA in PBS) and incubated with Zombie NIRTM (Biolegend, 1:100) for 20 min at room temperature, and then cells were stained with extracellular antibodies: anti-mouse CD3 FITC, anti-mouse CD8a APC, anti-mouse CD4 PerCP/Cy5.5, anti-mouse Ly-6G/Ly-6C (Gr-1) PE/Cy7, anti-mouse CD11b PE, anti-mouse CD 279 (PD-1) Brilliant Violet 421TM or anti-mouse CD45 Brilliant Violet 510TM (Biolegend). For intracellular staining, cells were stained using the FoxP3/T-bet/IFN-γ/granzymeB (GrzmB) staining buffer set (Biolegend) for fixation and permeabilization after completion of extracellular staining according to the manufacturer’s protocol. Cells were then stained with Biolegend’s anti-mouse FoxP3 PE, anti-mouse T-bet PE/Cy7, anti-mouse GrzmB FITC or anti-mouse IFNr bright purple 510TM. All data were collected on a flow cytometer (BD Biosciences, Canto II) and analyzed using FlowJo v10 software (Tree Star, Inc.)

### Immunofluorescence

Immunofluorescence uses color fluorescence channels to image specific target proteins through a fluorescence microscope. Deparaffinized tissue sections were treated with antigen recovery solution (made of citrate buffer, pH 6.0, T0050 Diapath). The tissue sections were then incubated with peroxidase blocking solution (S2023, Dako) for 15 min and with protein blocking (X0909, Dako) for 20 min. All sections were incubated with CD8 (ab4055, Abcam) antibody. The sections were incubated with GrzmB (NBP1-97525, Novus), IFN-γ (MAB285-sp, Novus) and T-bet (MAB5385, Novus) antibodies, respectively. The signal was counterstained using Cy3 conjugated goat anti-rabbit IgG (GB21303, Servicebio) and AlexaFluor^®^ 488 conjugated AffiniPure goat anti-mouse IgG (GB25301, Servicebio). The slides were scanned with a fluorescence microscope (NIKON ECLIPSE TI-SR) at a magnification of 90 times.

### Participants

Patients aged 18 to 70 years who were diagnosed with stage I–III gastric cancer were recruited and randomly divided into placebo group and propranolol group. In this trial, nine patients were assigned to propranolol and 20 to placebo. Patients were given propranolol or a placebo 1 week before surgery for gastric cancer, and tumor tissue was collected after surgery. Tumor proliferation, phosphorylation of the AKT/MAPK signaling pathway, and immunity levels were assessed using immunohistochemistry and immunofluorescence. The clinical trial was approved by the Ethics committee of Xiangya Hospital of Central South University (no. 201702049) and registered in the ClinicalTrial.gov (NCT03245554). All patients in this study signed informed consent, and strictly followed the clinical trial protocol.

### Statistical Analysis

Student t test was used to compare data between two groups and tumor growth statistics were calculated using two-way ANOVA with Tukey analysis using Graphpad Prism software. One-way all data are depicted as mean ± SEM.

## Results

### Propranolol Inhibits Tumor Growth in Mice

**Figure 1:**
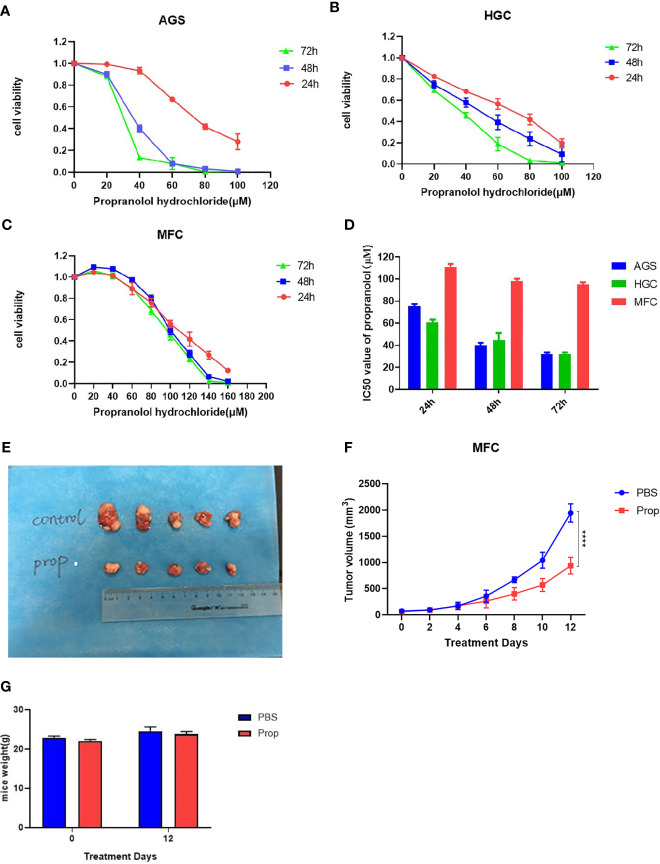
Propranolol inhibits tumor growth in mice. **(A-C)** Cell viability was determined in following propranolol concentrations: 0, 20, 40, 60, 80, 100, 120, 140 μM for 24, 48, and 72 h by MTS assay. **(D)** IC50 of propranolol on gastric cancer cell lines. **(E)** Tumors excised from MFC tumor mice, untreated mice (PBS group) and mice treated with propranolol (propranolol group). **(F)** The tumor volume were measured, and the results are shown. **(G)** The body weight of mice before or after propranolol treatment. Cell viability experiments were repeated for at least three times independently. ****P < 0.0001, N = 5 per group.

Propranolol significantly reduced *in vitro* cells viability of AGS and HGC in a concentration- and time-dependent manner (**Figures 1A, B** **)**. In MFC cell, propranolol inhibited cell proliferation in a concentration- but not time-dependent manner (**Figure 1C**). The data showed that the IC50 of propranolol on AGS and HGC cell lines were lower than MFC cell line (**Figure 1D** and **Table 1**). And propranolol did not inhibited the cell viability of normal intestinal epithelial cell line NCM460 (**Figure S1**). The therapeutic effect of propranolol on gastric cancer was then assessed in MFC tumor engrafted 615 mice. The mean tumor size of propranolol-treated group was smaller than the PBS group on day 12 in MFC tumor model (937.9 ± 55.95 mm^3^ vs. 1945 ± 70.38 mm^3^, unpaired *t* test, *P* < 0.0001, **Figures 1E, F** **)**. And there was no significant change in the body weight of the MFC mice before and after propranolol treatment (**Figure 1G**). Overall, these results indicated that propranolol can suppress the growth of gastric cancer.

**Table 1:** IC50 of different gastric cell lines.

Cell	Gastric cancer cell lines	
	Time	IC-50 value
ASG	24h	75.43
	48h	39.43
	72h	32.29
HGC	24h	60.86
	48h	44.51
	72h	32.29
MFC	24h	110.87
	48h	98.24
	72h	95.35

### Propranolol Inhibited Proliferation *In Vivo*

**Figure 2:**
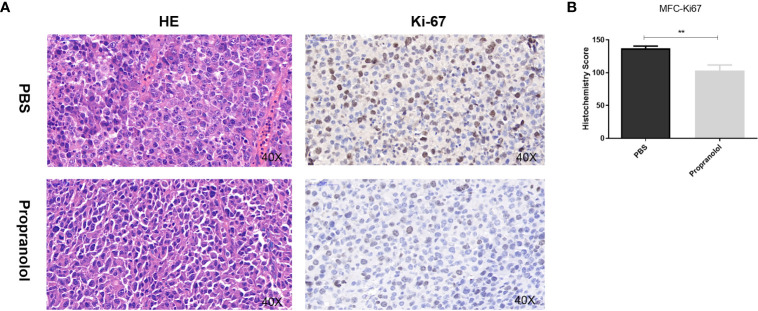
Propranolol inhibited proliferation *in vivo*. **(A)** HE staining for cell morphology in propranolol and PBS group. Ki-67 was assessed by immunohistochemistry assay. The picture was enlarged 40 times. **(B)** Quantification of Ki-67 staining. Results are presented as mean ± SEM. All experiments were repeated for three times independently. **P < 0.01, N = 5 per group.

Compared with PBS treatment, the number of nuclei with karyorhexis and karyolysis was greatly increased in propranolol treated group, suggesting that propranolol could promote cell necrosis in tumor (**Figure 2A**). We detected the expression of Ki-67 as a cell proliferation marker in tumor sections. The Ki-67 index was significantly decreased in MFC tumor treated with propranolol (136.9 ± 3.56 vs. 103.2 ± 8.59, *P* = 0.0067, **Figure 2B**).

### Propranolol Inhibited the AKT/MAPK Pathway *In Vivo*

**Figure 3:**
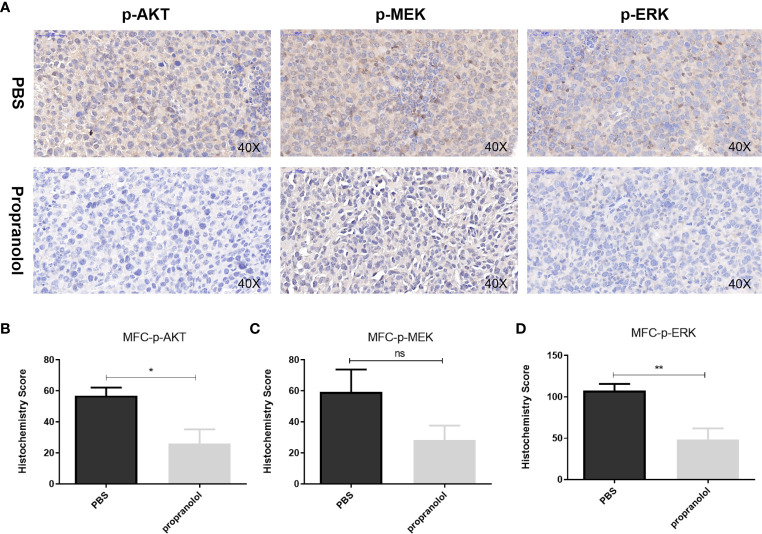
Propranolol inhibited the AKT/MAPK pathway *in vivo*. **(A)** p-Akt, p-MEK, and p-ERK were assessed by immunohistochemistry assay in tumor tissue both in propranolol and PBS groups. **(B–D)** Quantification of p-Akt, p-MEK, and p-ERK staining. Results are presented as mean ± SEM. All experiments were repeated for at least three times independently. ns, no significance; *P < 0.05, **P < 0.01, N = 5 per group.

As shown in **Figure 3**, the phosphorylation level of AKT (p-AKT) was reduced in the propranolol treated group compared with the PBS group in MFC tumor (56.82 ± 5.32 vs. 26.16 ± 9.1, *P* = 0.0196, **Figure 3B** **)**. p-ERK was significantly reduced in the propranolol treated mice (107.4 ± 8.3 vs. 48.2 ± 13.77, *P* = 0.0062, **Figure 3C**). Furthermore, p-MEK was slightly reduced by propranolol (59.28 ± 14.48 vs. 28.27 ± 9.42, *P* = 0.11, **Figure 3D**), although this was not statistically different. We also detected the expression of AKT/MAPK pathway following treatment of propranolol (corresponding IC50 concentration) for 24 h in AGS and HGC cell lines, the expression were slight reduced (**Figure S2**). These data revealed that propranolol could inhibit the growth of gastric cancer *in vivo* by inhibiting the phosphorylation of the AKT and MAPK pathways.

### Propranolol Did Not Change Tumor Infiltration of CD8^+^/CD4^+^ T Cells in MFC Mice

**Figure 4:**
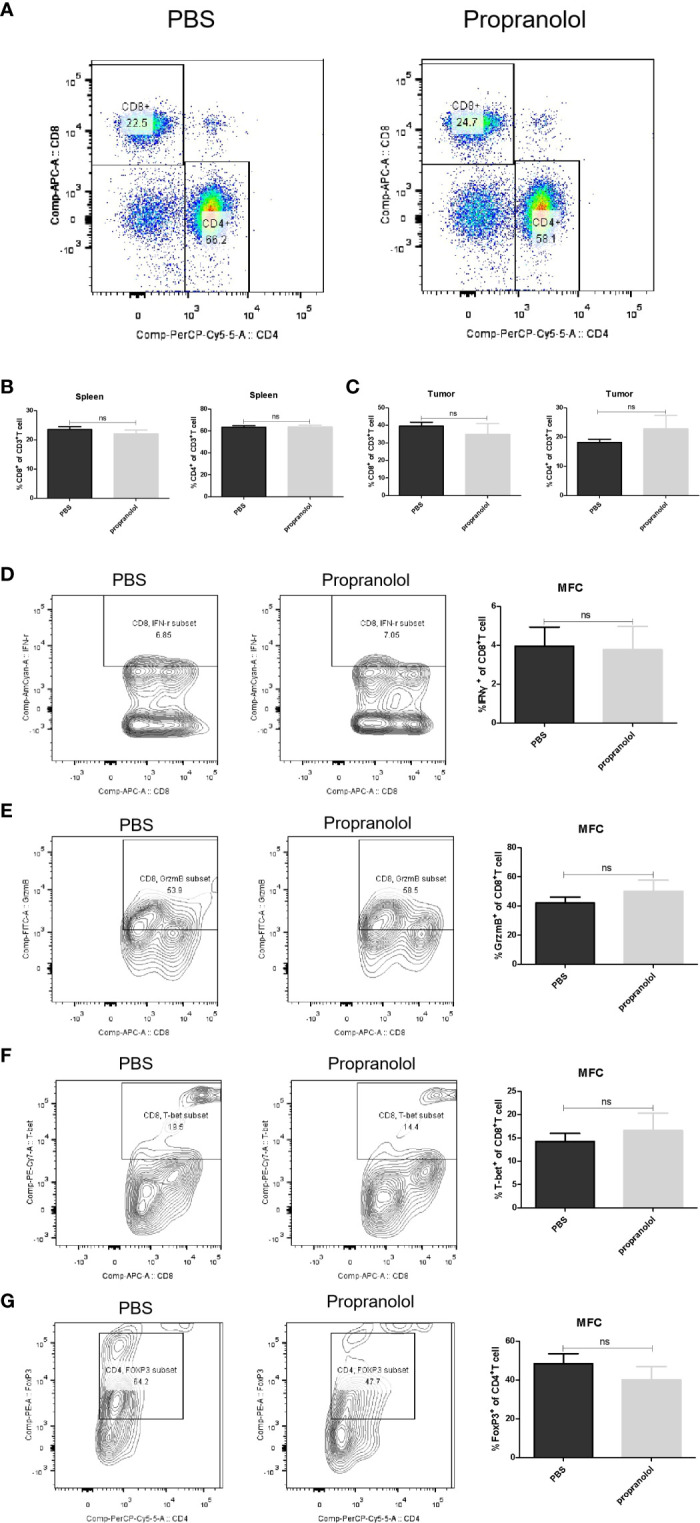
Propranolol effect on the immune cells in MFC mice. **(A)** the flow plots picture of CD8^+^, CD4^+^ T cells of CD3^+^ T cells in spleen of mice. **(B, C)** Quantification of CD8^+^ and CD4^+^ T cells in spleen and tumor tissue. **(D–G)** the contour plot and quantification of IFN-γ, GrzmB, T-bet of CD8^+^ T cells and CD4^+^FoxP3^+^ cells. Results are presented as mean ± SEM. NS, no significance; N = 5 per group.

The frequency of CD8^+^ and CD4^+^ T cells in tumor microenvironment (TME) and spleen was assessed *via* flow cytometry. As shown in **Figures 4A–C**, the frequency of CD8^+^ and CD4^+^ T cells were not changed with propranolol treatment from tumor tissue and spleen in mice. We also found that the expression of the effector molecules on CD8^+^ T cells in tumor tissue, including IFN-γ, GrzmB, T-bet, were all not significantly changed (**Figures 4D–F**). Similar result was reported for the ratio of CD4^+^FoxP3^+^ cells (**Figure 4G**). Data were shown in **Table 2**.

**Table 2:** Frequency of CD8+, CD4+ T cells and other cytokines in MFC spleen and tumor tissue.

Marker	MFC	
	Frequency%	*P*-value
CD8+ (spleen)	23.52 ± 0.99 *vs*. 22.00 ± 1.32	0.384
CD4+ (spleen)	63.40 ± 1.38 *vs*. 63.54 ± 1.88	0.954
CD8+ (tumor)	39.52 ± 2.28 *vs*. 34.86 ± 6.22	0.502
CD4+(tumor)	18.1 ± 1.08 *vs*. 22.80 ± 4.55	0.35
GrzmB	41.94 ± 4.19 *vs*. 50.06 ± 7.56	0.375
IFN-γ	3.95 ± 2.72 *vs*. 3.77 ± 1.20	0.911
T-bet	14.24 ± 1.73 *vs*. 16.58 ±3.79	0.59
FoxP3	48.46 ± 5.08 *vs*. 40.08 ± 6.90	0.357

### Propranolol Effects on the AKT/MAPK Pathway and CD8^+^ T Cell in Gastric Cancer Patients

**Figure 5:**
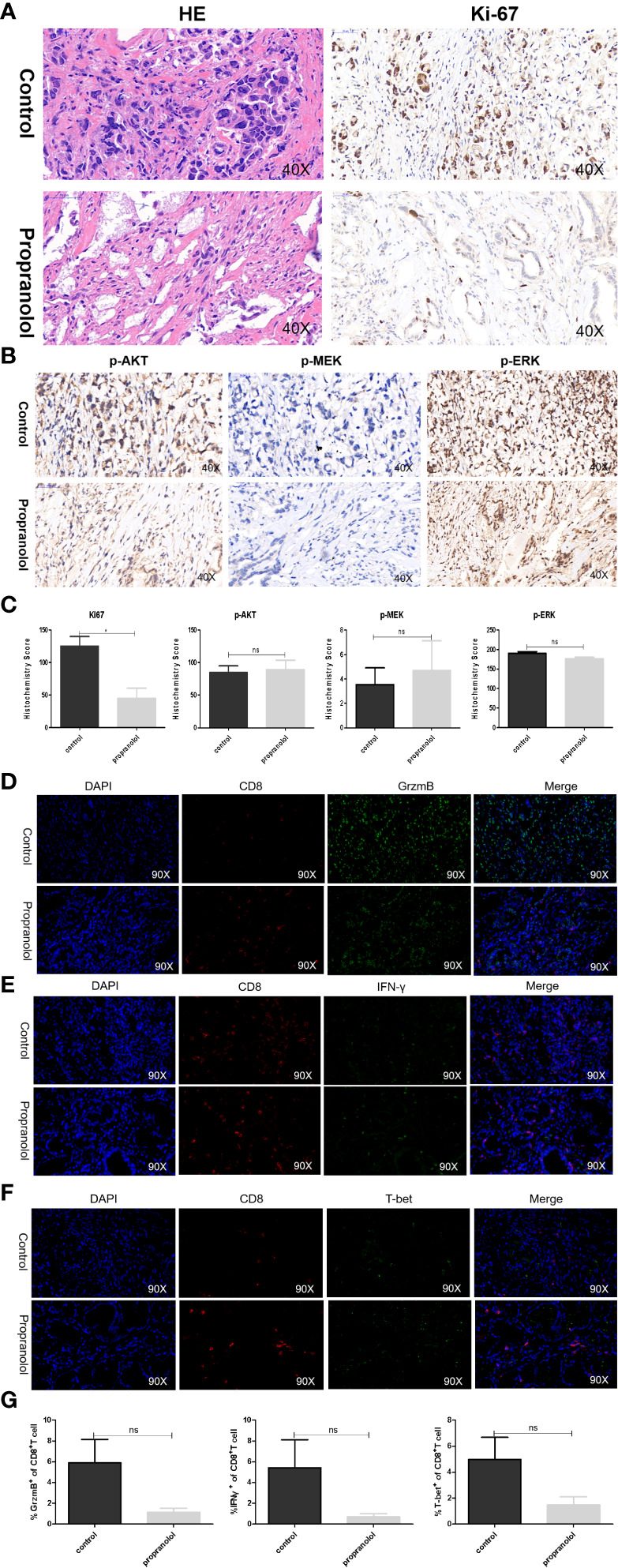
Propranolol effects on the AKT/MAPK pathway and CD8^+^ T cell in gastric cancer patients. **(A, B)** Ki-67, p-Akt, p-MEK, and p-ERK were assessed by immunohistochemistry assay in tumor tissue both in propranolol and PBS groups. **(C)** Quantification of Ki-67, p-Akt, p-MEK, and p-ERK staining. **(D–F)** Example pictures in tumor tissues using in the immunofluorescence panel, including GrzmB, IFN-γ, and T-bet in CD8^+^ T cells. The first column shows the cell nucleus in blue by 4′,6-diamidino-2-phenylindole (DAPI); the next column shows the presence of CD8^+^ T cells in red. The third column shows the factor of interest in green, and the final column shows the merged image of the three channels. **(G)** Quantification of the expression of GrzmB, IFN-γ, and T-bet in CD8^+^ T cells populations. N = 17 in control group and N = 4 in propranolol group. Results are presented as mean ± SEM. ns, no significance; *P < 0.05.

The basic characteristics for 29 gastric cancer patients are shown in **Table 3**. Patients received propranolol or placebo for 1 week before surgical resection. The expression of Ki-67 was reduced in patients receiving propranolol treatment than placebo (44.84 ± 15.60 vs 125.27 ± 14.76 in placebo; **Figure 5A**). However, the phosphorylated AKT, MEK, ERK were not significant different between two group (**Figures 5B, C** **)**. Furthermore, the expression of IFN-γ, GrzmB, T-bet on CD8^+^ T cell were numerically lower in the propranolol treated patients, although this did not reach statistical significance (**Figures 5D–G**). Specific data were shown in **Tables 4**, **5**.

**Table 3:** The characteristics of gastric cancer patients.

	Gastric cancer (*n)*	
	Control	Propranolol
Age		
<60	9	4
≥60	11	5
Gender		
Female	8	2
Male	12	7
Tumor stage		
I	1	1
II	6	5
III	13	3

**Table 4:** p-AKT, p-ERK, and p-MEK values in gastric cancer patient.

	Gastric cancer patient	
Protein	H-Score	*P*-value
Ki-67	125.27±14.76 VS. 44.84±15.60	0.02
p-AKT	84.82 ± 10.42 *VS. * 89.45 ± 14.06	0.841
p-ERK	190.21 ± 4.47 *vs.* 176.26 ± 4.19	0.161
p-MEK	3.54 ± 1.36 *vs.* 4.70 ± 2.42	0.704

**Table 5:** Expression of GrzmB, IFN-γ, PD-1, and T-bet on CD8^+^ T cell.

	Gastric cancer patient	
Marker	% of CD8+ T cells	*P*-value
GrzmB	5.90 ± 2.26 *vs.* 1.15 ± 0.38	0.331
IFN-γ	5.40 ± 2.72 *vs.* 0.70 ± 0.31	0.421
T-bet	4.98 ± 1.71 *vs.* 1.48 ± 0.63	0.344

## Discussion

This study elucidated that propranolol had antiproliferative activity in both mice model and gastric cancer patients although it could not increase tumor infiltration of CD8^+^ T cells. Several lines of evidences have proved that β_2_-ARs are abundantly expressed on the gastric cancer cells (12–15). Inhibition of β_2_-ARs can reduce the concentration of cAMP and inhibit the activation of protein kinase A (PKA), thereby affecting the expression of downstream transcription factors, such as CREB and NF-KB, leading to inhibition of tumor cell proliferation. Zhou and colleagues found that propranolol can induce melanoma cell apoptosis and inhibit the proliferation *in vitro* and *in vivo* by inhibiting the phosphorylation of AKT, BRAF, MEK1/2, and ERK1/2 (8). Liao et al. also found that propranolol inhibited colorectal cancer proliferation by regulating the AKT and MAPK pathways (7). Previous studies demonstrated that propranolol can down-regulate the expression of CyclinD1, leading to G1/S arrest (16, 17). Our results showed that propranolol decreased the expression of Ki-67 and the phosphorylation of AKT, MEK, and ERK which means that propranolol can inhibit the growth of gastric cancer. And our colleague found that propranolol can affect intracellular pH in bladder cancer cell lines, maybe this is a reason why propranolol can affect the AKT/MAPK signaling pathway.

Consistent with mouse tumor models, the expression of Ki-67 was significantly reduced in propranolol treated gastric cancer patients’ samples; however, the phosphorylation of AKT and ERK was not. The length of propranolol treatment, which was only 1 week prior to surgery in patients, could be the major explanation for the lack of change for AKT/ERK phosphorylation. Longer treatment of propranolol could lead to more apparent differences in p-AKT and p-ERK in gastric cancer samples.

T cells expresses β-adrenergic receptors (AR) which could be activated by norepinephrine (NE) released from sympathetic nerve endings. Stimulation of β-ARs on T cells increases cAMP and PKA and inhibits T cell proliferation (18). Our group previously demonstrated that propranolol activated autologous CD8^+^ T cells in both colorectal cancer mouse models and patients by blocking the activation of β-ARs. Inconsistent with this finding, the expression of GrzmB/IFN-γ/T-bet in the CD8+ T-cell population were not significantly altered in gastric cancer. It is known that T cells also express muscarinic and nicotinic acetylcholine (ACh) receptors which can be activated by Ach released from efferent cholinergic nerves (19), while stomach is mainly innervated by the vagus nerve (VN), which is comprised of both sensory and preganglionic parasympathetic fibers that release Ach in gastric tumors. Ach acting on α7 nicotinic receptors (nAChRs) inhibits cytokine production by T lymphocytes (20). Blocking β-ARs could relieve the inhibition on T lymphocytes by NE, however, Ach released by vagus nerve could still modulate T lymphocytes which indicated a more complex immune environment in gastric cancer.

In summary, these results revealed that propranolol could inhibit gastric cancer growth but failed to activate the CD8^+^ T cell in mice and patients. In short, propranolol plays an important role in the treatment of tumors, opening a new chapter for this old drug. More clinical trials are needed to prove the anti-tumor effect of propranolol. Two clinical trials including one for recruiting gastric cancer patients in a single-arm design (NCT04005365) and a randomized controlled clinical trial in bladder cancer patients (NCT04493489) were designed to further explore the anti-tumor effect of propranolol.

## Data Availability Statement

The original contributions presented in the study are included in the article/**Supplementary Material**. Further inquiries can be directed to the corresponding authors.

## Ethics Statement

The patients/participants provided their written informed consent to participate in this study. The animal study was reviewed and approved by Animal Ethics Committee of Central South University.

## Author Contributions

QH and PL conceived and operated experiments, and wrote manuscripts. WL, JH, CC, YZ, and YW helped with the experiment. LC, KS, JL, WZ, and QL gave instructions for the experiment. HM and YH provided suggestions on experimental design, experimental process, experimental results and manuscript writing. All authors contributed to the article and approved the submitted version.

## Funding

This study was supported by the National Natural Science Foundation of China (grants 81673517 and 81773821); Chinese National Major Project for New Drug Innovation (grant 2019ZX09201-002-006), the National Key Research and Development Program (2016YFC0905000), the research on precision colorectal surgery based on parallel AR auxiliary system Key R&D Program of Hunan Province (2018SK2129, China), the key project of health commission of Hunan Province (202113010141). Natural Science Foundation of Hunan Province, China (2019JJ40477).

## Conflict of Interest

HM is on the board of directors for Cancer Genetics Inc and on a Speakers Bureau for Genentech. He is one of the founders of Interpares Biomedicine and Clariifi and a consultant to Pharmazam and eviCORE Health Solutions.

The remaining authors declare that the research was conducted in the absence of any commercial or financial relationships that could be construed as a potential conflict of interest.
