# Supplementary material for: Clinical Use of Propranolol Reduces Biomarkers of Proliferation in Gastric Cancer
Source: Front Oncol. 2021 Apr 26;11:628613. doi: 10.3389/fonc.2021.628613 (PMC8108985; doi:10.3389/fonc.2021.628613)
Supplement: Supplementary file 1 [file DataSheet_1.pdf]

Supplementary materials

Figure S1: Cell viability of NCM460. Cell viability was determined in following propranolol concentrations: 0 μM, 20 μM, 40 μM, 60 μM, 80 μM, 100 μM for 24h, 48h and 72h by MTT assay.

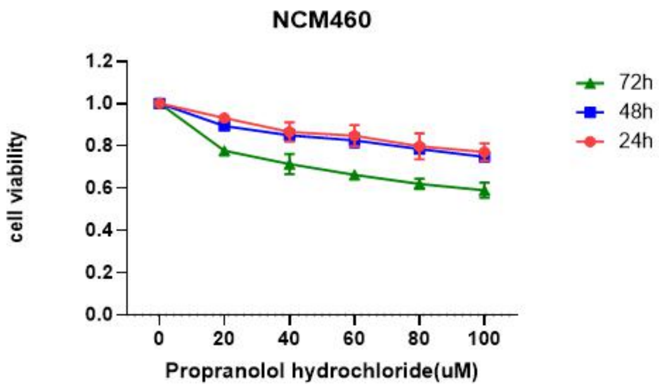

Figure S2: Propranolol inhibited proliferation by inhibiting AKT/MAPK pathway. A-B, The expression of GADPH, AKT, MEK, ERK, p-AKT, p-MEK, p-ERK following treatment of propranolol (corresponding IC50 concebtration) for 24h in AGS and HGC cell lines. E-F, Quantification of each protein. Results are presented as mean±SEM. All experiments were repeated for at least three times independently.

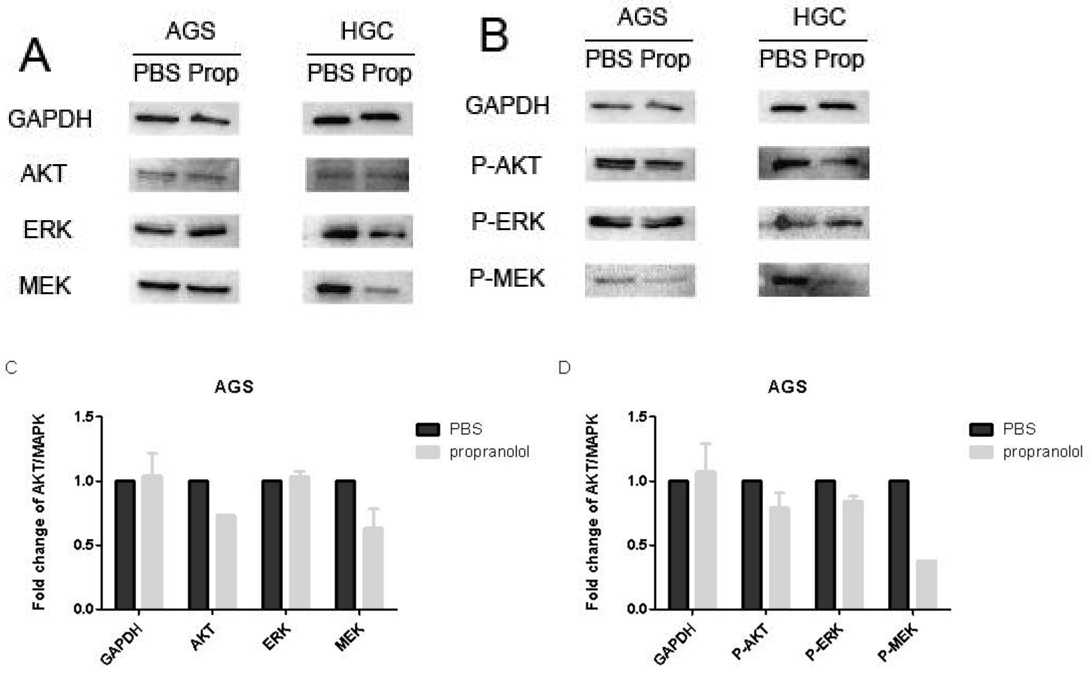

E

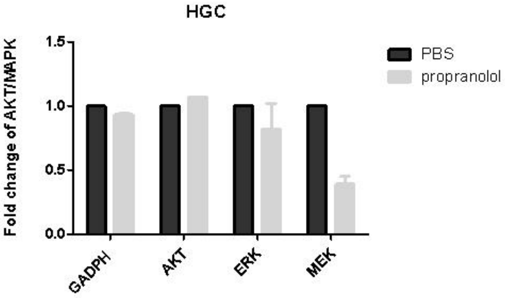
HGC
Fold change of AKT/MAPK
1.5
1.0
0.5
0.0
GADPH
AKT
ERK
MEK
PBS
propranolol

F

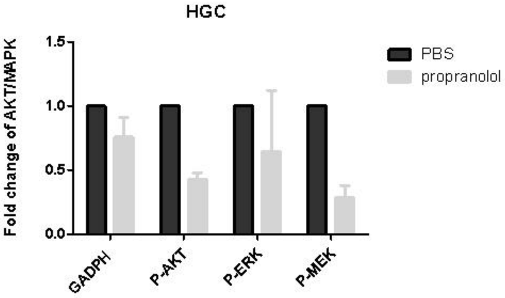
HGC
Fold change of AKT/MAPK
1.5
1.0
0.5
0.0
GADPH
P-AKT
P-ERK
P-MEK
PBS
propranolol
